# Confirmatory Factor Analysis of the 12-Item Center for Epidemiologic Studies Depression Scale among Blacks and Whites

**DOI:** 10.3389/fpsyt.2016.00178

**Published:** 2016-11-07

**Authors:** Shervin Assari, Ehsan Moazen-Zadeh

**Affiliations:** ^1^Department of Psychiatry, University of Michigan, Ann Arbor, MI, USA; ^2^Center for Research on Ethnicity, Culture and Health, School of Public Health, University of Michigan, Ann Arbor, MI, USA; ^3^Psychiatric Research Center, Roozbeh Hospital, Tehran University of Medical Sciences, Tehran, Iran; ^4^Mental Health Research Center, Tehran Psychiatric Institute, School of Behavioral Sciences and Mental Health, Iran University of Medical Sciences, Tehran, Iran

**Keywords:** racial groups, Blacks, African-Americans, Whites, depressive symptoms, Center for Epidemiologic Studies Depression, reliability

## Abstract

**Background:**

The Center for Epidemiologic Studies Depression (CES-D) scale is one of the most widely used tools to measure depressive symptoms in epidemiological studies. Given the importance of cross-racial measurement equivalence of the CES-D scale for research, we performed confirmatory factor analysis (CFA) of the 12-item CES-D in a nationally representative sample of Black and White adults in the United States.

**Methods:**

We used data from the National Survey of American Life (NSAL), 2001–2003. A total number of 3570 Blacks (African-Americans) and 891 non-Hispanic Whites were included in the present study. CFA was carried out on the 12-item CES-D scale using multi-group structural equation modeling.

**Results:**

For both Blacks and Whites, the best fitting model was found to be the 3-factor model, indicating invariance of factor structure between Blacks and Whites. A statistically different fit of the models with and without constraints indicated lack of invariance of factor loadings between Blacks and Whites. Some of the positive (i.e., “*as good*” and “*hopeful*”) and interpersonal (i.e., “*people were unfriendly*”) items showed poor loadings, even in the 3-factor solution that allowed separate domains for positive affect, negative affect, and interpersonal problems. Despite the good fit of our final model, more items (i.e., “*as good*,” “*hopeful*,” “*keeping mind*,” and “*everything effort*”) had poorer loadings in Blacks than Whites (i.e., “*as good*”).

**Conclusion:**

There is invariance in factor structure but lack of invariance in factor/item loadings between Blacks and Whites. These findings have implications for cross-racial studies of depressive symptoms using CES-D scale among Blacks and Whites. Further research is warranted to scrutinize the role of socioeconomics and culture in explaining the lack of invariance of the CES-D scale between Blacks and Whites.

## Introduction

Depression accounts for the largest portion of global burden of mental disorders (1). Considering that cross-ethnic studies have estimated the prevalence of depression ranging from 1.5 to 32% across ethnicities (2–5), accurate measurement of depression as well as depressive symptoms across racial and ethnic groups has attracted special attention (6–13). The Center for Epidemiologic Studies Depression scale (CES-D) is one of the most commonly used tools for measuring depressive symptoms in epidemiological (13) as well as clinical (14) studies. Since the introduction of the original 20-item CES-D scale, which evaluates major domains of depressive symptoms (i.e., 7 items of negative affect, 4 items of positive affect, 7 items of somatic symptoms, and 2 items of interpersonal problems), different abbreviated versions with variable number of items have been applied in epidemiological studies, (13–25) among them the widely used 20-item and 10-item CES-D scales (13, 19). The abbreviated versions were mainly developed to lower respondent burden and accelerate administration and scoring of the scale (18). They mostly reduced on the number of items for negative affect and somatic symptoms while sparing items for interpersonal problems. In this way, a 12-item version of the CES-D scale has also been developed and used in some major epidemiological studies including the National Survey of American Life (NSAL) (22, 25).

Measurement equivalence of the CES-D scale between Blacks and Whites has been the focus of several studies since its first application (13, 15–23). For example, some studies have reported lower overall reliability of the scale for measurement of depressive symptoms (17) but higher reliability of negative affect and interpersonal problem domains (15) of the 20-item CES-D scale in Blacks compared to Whites. There are also studies showing invariance of factor structure for both 10-item (16) and 20-item (17) CES-D scale across racial and ethnic groups. Studies that have found a lack of invariance for some of the CES-D scale items have concluded that response to some of the CES-D scale items may be group specific (16).

In a recent longitudinal study, using Americans’ Changing Lives data, despite the high reliability of an 11-item version of CES-D scale in both Blacks and Whites, CES-D scale total score was predictive of depression 15 years later in Whites but not Blacks based on diagnosis by Composite International Diagnostic Interview (CIDI). In this study, the CES-D scale item “*people disliked me*” loaded better into the positive affect factor in Blacks but better into the interpersonal problems factor in Whites (24). In another cross-sectional study on NSAL data using the 12-item CES-D scale, CES-D scale negative affect and interpersonal problem domains were more strongly associated with CIDI-based major depressive disorder (MDD) in Blacks compared to Whites (25).

In a study on older adults using the 20-item CES-D scale, Blacks endorsed disproportionately higher interpersonal problem items than Whites. That study also found higher loadings for interpersonal problem items in the single factor solution for the CES-D scale (26). In another study that compared low socioeconomic Blacks and a sample of Blacks and Whites based on the 20-item CES-D scale, different item loadings were found between Blacks and Whites. While items better loaded into negative affect and interpersonal problem domains for Blacks, the items better loaded into positive affect domain for Whites (27). Other researchers found similar patterns when comparing Black and White samples stratified on gender using the 20-item CES-D scale (28). Comparing sociodemographically matched Black and White pregnant women, Canady et al. found that the positive item “*happy*” was the only item among the 20 items with different loadings between Blacks and Whites (29). This finding was similar to what Moazen-Zadeh and Assari found recently investigating the 11-item version of the CES-D scale (24).

Early investigations into factor structure of affect in a predominantly White sample have indicated positive and negative affect as two distinct dimensions (30). Although high negative affect and low positive affect are major components of depression presentation (31–33), individuals may endorse varying levels of these dimensions simultaneously (32, 33).

Culture may alter how individuals endorse or express positive and negative affect (34, 35). The Black–White gap in endorsement of positive and negative affect items in self-report measures has been attributed to cultural differences (24, 29). In more collectivist and inter-connected cultures, specifically Blacks and Koreans, individuals have a lower tendency for endorsement of positive affect items compared to Whites (34, 35). In a recent study, the association between depression and hopelessness was stronger for Whites than Blacks, suggesting that even when depressed, Blacks maintain higher levels of hope than Whites (32).

Concerning the factor structure of the CES-D scale, a recent study on the concordance between the CES-D scale and the CIDI-based diagnosis of depression found the 3-factor model as the best solution for the 11-item CES-D scale (24). Previous studies have indicated that the 4-factor model (i.e., positive affect, negative affect, somatic complaints, interpersonal problems) may be the best solution for the 20-item CES-D scale with very high overall fit in Blacks and Whites (27–29, 36). A meta-analysis, however, found inconsistencies in the results of previous studies on the factor structure of the CES-D scale and concluded that the 4-factor model may not be suited to all racial groups (37). In addition, literature has indicated that somatic complaints correlate better with negative affect rather than positive affect, and this difference is more prominent in specific racial groups such as Blacks (38–40).

A growing body of evidence has indicated some major Black–White differences in socioeconomic and physical health correlates of depressive symptoms in the general population. For instance, Blacks and Whites differ in how depressive symptoms (CES-D score) correlate with education, chronic medical conditions, body mass index, and mortality (41–43). To give examples, a higher depressive symptoms score predicted increased risk of incident chronic disease (41, 44), as well as all-cause (42) and cause specific (43) mortality for Whites but not Blacks. It is still unknown to what degree these findings can be attributed to measurement bias; thus, there is a need to test measurement invariance for the CES-D scale between Blacks and Whites.

In a recent study, Assari and Moazen-Zadeh compared Blacks and Whites for the associations between positive affect, negative affect, and interpersonal problems measured using the 12-item CES-D and CIDI-based depressive diagnoses (i.e., lifetime MDD, lifetime major depressive episode (MDE), 12-month MDE, 30-day MDE, and 30-day major depressive disorder with hierarchy (MDDH)). For most CIDI-based depressive diagnoses, there was a positive and significant interaction between race and negative affect domain, as well as interpersonal problems domain, indicating stronger associations for Blacks compared to Whites. However, the CES-D scale total score and positive affect did not interact with race for CIDI-based depressive diagnoses. Authors concluded that these differences may be due to higher depressive symptoms among Blacks who endorse the CIDI criteria for the clinical depressive disorder considering that Blacks have a lower tendency to receive treatment for depression (25).

The current confirmatory factor analysis (CFA) compared Blacks and Whites for a 12-item CES-D scale factor structure and item loadings, using NSAL dataset which includes a large and nationally representative sample of Blacks and Whites, and therefore provides the researchers with an exceptional opportunity to investigate the cross-racial measurement properties of the CES-D scale (22, 25).

## Materials and Methods

We used data from the NSAL, 2001–2003. The NSAL has been the most comprehensive study of mental health on Blacks and proportionately sampled Whites with a household probability sampling of adolescents (13–17 years old) and adults (older than 17 years old) from 48 conterminous states (45, 46). Detailed measures of health, social conditions, distress, as well as psychosocial protective/risk factors are included in this study (45). Detailed information on the NSAL study is available in the literature (45–47).

### Participants

This study included 891 non-Hispanic Whites (Whites) and 3570 African-Americans (Blacks) who participated in the NSAL study. We did not include Caribbean Blacks (*n* = 1623). Thus, our participants were Blacks with no Caribbean ancestral ties (48). In more detail, respondents were asked about their racial group as well as their parents’ racial group. Also, they were asked about the state and country of their birth as well as their parents’ birth place. Furthermore, data were collected on their age and reason for migration if any. Consequently, those individuals self-identifying as Black were considered Caribbean Black if any of the following conditions existed: West Indian or Caribbean descent; from Caribbean area country; parents or grandparents born in a Caribbean area country. In the NASL study, Blacks and Whites were sampled from urban and rural areas with the same contexts and geographical areas for both race groups in order to optimize the sample for comparative analyses (45). Detailed information on sampling is available in the literature (45–48).

### Interview

Interviews were carried out in English whether face-to-face (86%) or via telephone (14%) with a 70.7 and 69.7% response rate for Blacks and Whites, respectively.

### Measures

#### Depressive Symptoms

An abbreviated 12-item version of the CES-D scale was used which evaluates major domains of depressive symptoms including negative affect (e.g., I felt depressed), positive affect (e.g., I was happy), somatic symptoms (e.g., my sleep was restless), and interpersonal problems (e.g., people were unfriendly). Acceptable validity and reliability of the CES-D scale have been confirmed in several studies (18, 19, 49). The CES-D scale items are listed in Table A1 in Appendix.

#### DSM-Based Diagnoses of Depression

Five DSM-based diagnoses of depression including lifetime MDE, 12-month MDD w/hierarchy, 12-month MDE, 30-day MDD w/hierarchy, and 30-day MDE were measured using a modified version of the World Mental Health CIDI. The CIDI is a fully structured diagnostic interview and evaluates a wide range of Diagnostic and Statistical Manual-IV (DSM-IV) mental disorders. The CIDI has been used reliably in the World Mental Health project (8, 9, 25).

### Ethics

The NSAL study was approved by Institutional Review Board of the University of Michigan in accordance with the Code of Ethics of the World Medical Association (Declaration of Helsinki, Edinburgh 2000 revision). Informed consent was obtained from all participants.

### Statistical Note

In this study, we performed our descriptive univariate analysis in the SPSS statistical package (IBM Corp, Armonk, NY, USA). Pearson correlation test was used to assess bivariate correlations between CES-D scale items and CES-D total score. The correlation between psychiatric diagnoses and CES-D scores were assessed by Spearman’s rho. We used Amos 20 (IBM Corp, Armonk, NY, USA) for CFA. The *p* < 0.05 was considered as statistically significant.

For CFA, we used multi-group structural equation modeling (SEM) to estimate the fit of 1-, 2-, and 3-factor models between Blacks and Whites. In our multi-group analysis, group was defined based on race (50).

To handle missing data, the Amos uses Full Information Maximal Likelihood (FIML) (51, 52). As a method frequently used in SEM, FILM estimates parameters by maximizing the likelihood function of the incomplete data rather than imputing the missing data directly. The model fits were assessed by examining the chi-square statistic, the comparative fit index (CFI), and the root mean square error of approximation (RMSEA). A non-significant chi-square statistic, a CFI above 0.95, and a RMSEA value of 0.05 or less are indicators of a good fitting model to the data (53). In case of chi-square/degrees of freedom (in AMOS defined as CMIN/df) fit index, there is no consensus regarding an acceptable ratio and recommendations range from 2 to 5 (54).

To compare fit indices of various nested models with and without constraints, different number of factors, and error covariance, we applied chi*-*square difference tests. The constraints were added to all item loadings rather than covariance between the factors. For appropriate loading, 0.50 was considered as the required threshold.

We were interested in behaviors of items and factors with and without imposing constraints rather than finding the best fitting models. In other words, we wanted to know how each individual item behaves (across models) for Whites and Blacks. Therefore, we ran six models. *Model 1* was 1-factor model of CES-D, without constraints. *Model 2* was 1-factor model of CES-D, with constraints. *Model 3* was 2-factor model of CES-D, without constraints. *Model 4* was 2-factor model of CES-D, with constraints. *Model 5* was 3-factor model of CES-D, without constraints. *Model 6* was 3-factor model of CES-D, with constraints. All models allowed for error covariance. Comparison of fit indices between models with the same number of factors with and without constraints (*Model 1* vs. *Model 2, Model 3* vs. *Model 4, Model 5* vs. *Model 6*) were indicative of invariance or lack of invariance of the loadings between Blacks and Whites. If the fit significantly dropped with adding the constraints, it would suggest that the loadings are not identical across groups, indicating lack of invariance based on race.

## Results

### Descriptive Statistics

Table 1 shows descriptive statistics for Black and White participants. While both Blacks and Whites had similar distribution of gender, Whites were older than Blacks. Blacks also had lower income than Whites. In addition, more Blacks were sampled from the Midwest, compared to Whites.

**Table 1:** **Demographic data among non-Hispanic Whites and Blacks**.^a^

	Race		
	Blacks *n* (%)	Whites *n* (%)	Total *n* (%)
Gender			
Male	1914 (44.42)	372 (47.26)	2286 (45.87)
Female	3277 (55.58)	519 (52.74)	3796 (54.13)
Region			
Northeast	1546 (18.01)	107 (22.67)	1653 (20.56)
Midwest	607 (17.94)	83 (7.96)	690 (12.91)
South	2786 (54.60)	609 (54.60)	3395 (54.48)
West	252 (9.20)	92 (14.76)	344 (12.06)
	**Mean (SE)**	**Mean (SE)**	**Mean (SE)**
Age	42.24 (0.49)	44.98 (0.31)	43.61 (0.69)
Income	41,863.62 (846.25)	46,831.74 (1545.97)	36,823.00 (654.60)

*^a^Weights have been applied*.

Table 2 provides correlation matrices between CES-D scale items, CES-D scale total score, and DSM-based diagnoses among Blacks and Whites. The CES-D scale items showed better correlations with each other and with DSM-based diagnoses among Blacks, compared to Whites.

**Table 2:** **Correlation matrix of CES-D scale items with CES-D total score, and DSM-based diagnoses among Blacks and Whites**.

	1	2	3	4	5	6	7	8	9	10	11	12	13	14	15	16	17	18
1 CES-D 1	1.000	0.101**	0.225**	0.041*	0.232**	0.136**	0.295**	0.137**	0.275**	0.178**	0.155**	0.165**	0.453**	0.089**	0.089**	0.119**	0.065**	0.080**
2 CES-D 2	−0.010	1.000	0.375**	0.201**	0.096**	0.286**	0.214**	0.211**	0.186**	0.231**	0.231**	0.310**	0.525**	0.130**	0.128**	0.151**	0.110**	0.130**
3 CES-D 3	0.196**	0.399**	1.000	0.232**	0.180**	0.424**	0.421**	0.248**	0.356**	0.488**	0.336**	0.428**	0.704**	0.265**	0.282**	0.332**	0.240**	0.275**
4 CES-D 4	0.020	0.366**	0.490**	1.000	0.000	0.196**	0.095**	0.171**	0.050**	0.170**	0.182**	0.186**	0.435**	0.059**	0.057**	0.078**	0.056**	0.070**
5 CES-D 5	0.409**	0.124**	0.250**	0.145**	1.000	0.070**	0.305**	0.079**	0.273**	0.099**	0.113**	0.132**	0.422**	0.053**	0.076**	0.085**	0.063**	0.068**
6 CES-D 6	0.070	0.272**	0.420**	0.363**	0.149**	1.000	0.290**	0.236**	0.235**	0.338**	0.256**	0.338**	0.586**	0.185**	0.203**	0.232**	0.166**	0.185**
7 CES-D 7	0.361**	0.188**	0.429**	0.249**	0.572**	0.216**	1.000	0.179**	0.574**	0.299**	0.221**	0.278**	0.626**	0.181**	0.204**	0.244**	0.150**	0.181**
8 CES-D 8	0.030	0.218**	0.261**	0.267**	0.098*	0.286**	0.089*	1.000	0.130**	0.241**	0.437**	0.231**	0.496**	0.062**	0.088**	0.106**	0.046**	0.049**
9 CES-D 9	0.386**	0.173**	0.401**	0.210**	0.532**	0.220**	0.727**	0.070	1.000	0.263**	0.203**	0.259**	0.555**	0.212**	0.248**	0.280**	0.175**	0.200**
10 CES-D 10	0.185**	0.205**	0.428**	0.261**	0.184**	0.311**	0.253**	0.271**	0.238**	1.000	0.372**	0.386**	0.589**	0.204**	0.233**	0.275**	0.149**	0.178**
11 CES-D 11	0.142**	0.301**	0.368**	0.287**	0.218**	0.270**	0.246**	0.490**	0.227**	0.487**	1.000	0.361**	0.566**	0.158**	0.142**	0.184**	0.089**	0.111**
12 CES-D 12	0.106*	0.379**	0.443**	0.420**	0.245**	0.378**	0.309**	0.226**	0.338**	0.376**	0.341**	1.000	0.598**	0.192**	0.195**	0.221**	0.169**	0.176**
13 CES-D total	0.420**	0.520**	0.729**	0.595**	0.572**	0.577**	0.666**	0.456**	0.648**	0.577**	0.598**	0.648**	1.000	0.263**	0.286**	0.340**	0.219**	0.253**
14 MDE (lifetime)	0.060	0.189**	0.298**	0.155**	0.152**	0.171**	0.247**	0.070	0.217**	0.219**	0.168**	0.211**	0.306**	1.000	0.642**	0.711**	0.402**	0.447**
15 MDD (12 months)	0.105*	0.167**	0.331**	0.218**	0.177**	0.172**	0.246**	0.147**	0.204**	0.281**	0.233**	0.252**	0.357**	0.622**	1.000	0.903**	0.626**	0.559**
16 MDE (12 months)	0.105*	0.198**	0.362**	0.245**	0.195**	0.202**	0.268**	0.176**	0.239**	0.326**	0.251**	0.281**	0.401**	0.644**	0.965**	1.000	0.565**	0.628**
17 MDD (30 days)	0.040	0.101*	0.192**	0.116**	0.109*	0.130**	0.156**	0.070	0.116**	0.204**	0.164**	0.138**	0.216**	0.383**	0.616**	0.595**	1.000	0.901**
18 MDE (30 days)	0.040	0.128**	0.231**	0.150**	0.126**	0.159**	0.189**	0.091*	0.155**	0.251**	0.171**	0.172**	0.263**	0.405**	0.580**	0.628**	0.947**	1.000

*CES-D, Center for Epidemiologic Studies Depression; MDE, major depressive episode; MDD, major depressive disorder*.

*Blacks upper diagonal, Whites lower diagonal*.

*p < 0.05.

**p < 0.01.

### Factor Structure

**Figure 1:**
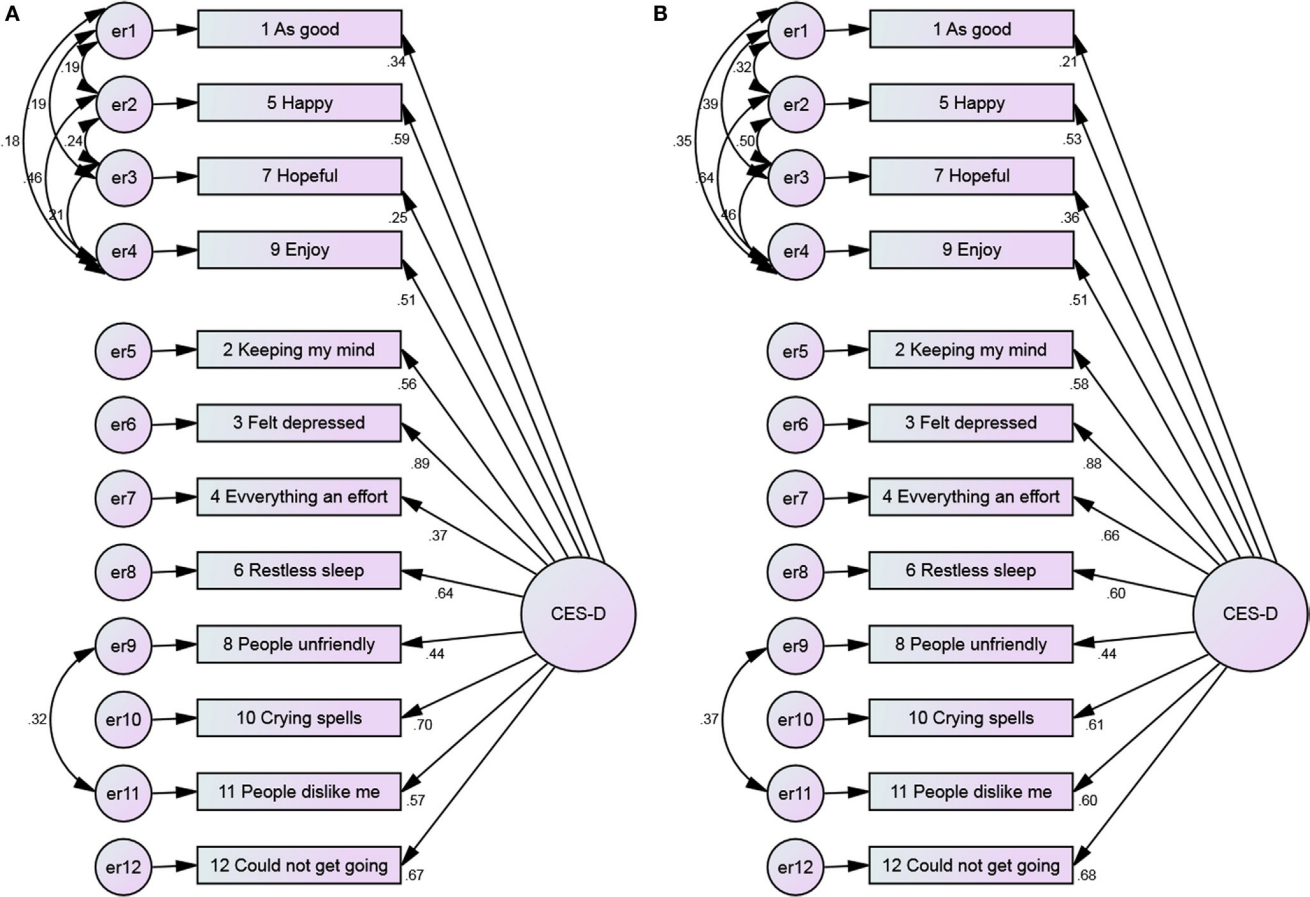
**The 1-factor model of the CES-D 12, with error covariance and no constraints among Blacks and Whites**. **(A)** Blacks and **(B)** Whites.

**Figure 2:**
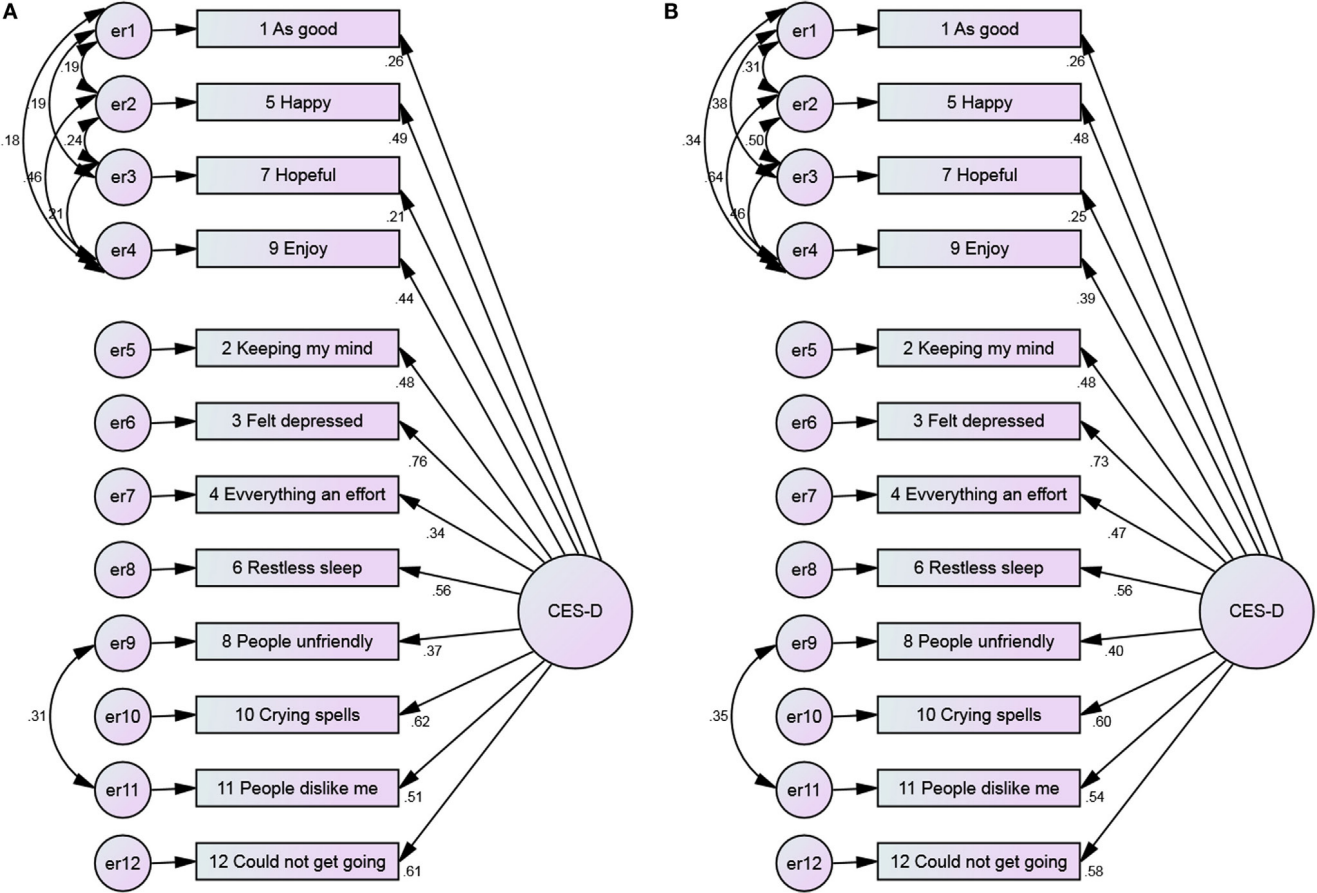
**The 1-factor model of the CES-D 12, with error covariance and constraints among Blacks and Whites**. **(A)** Blacks and **(B)** Whites.

**Figure 3:**
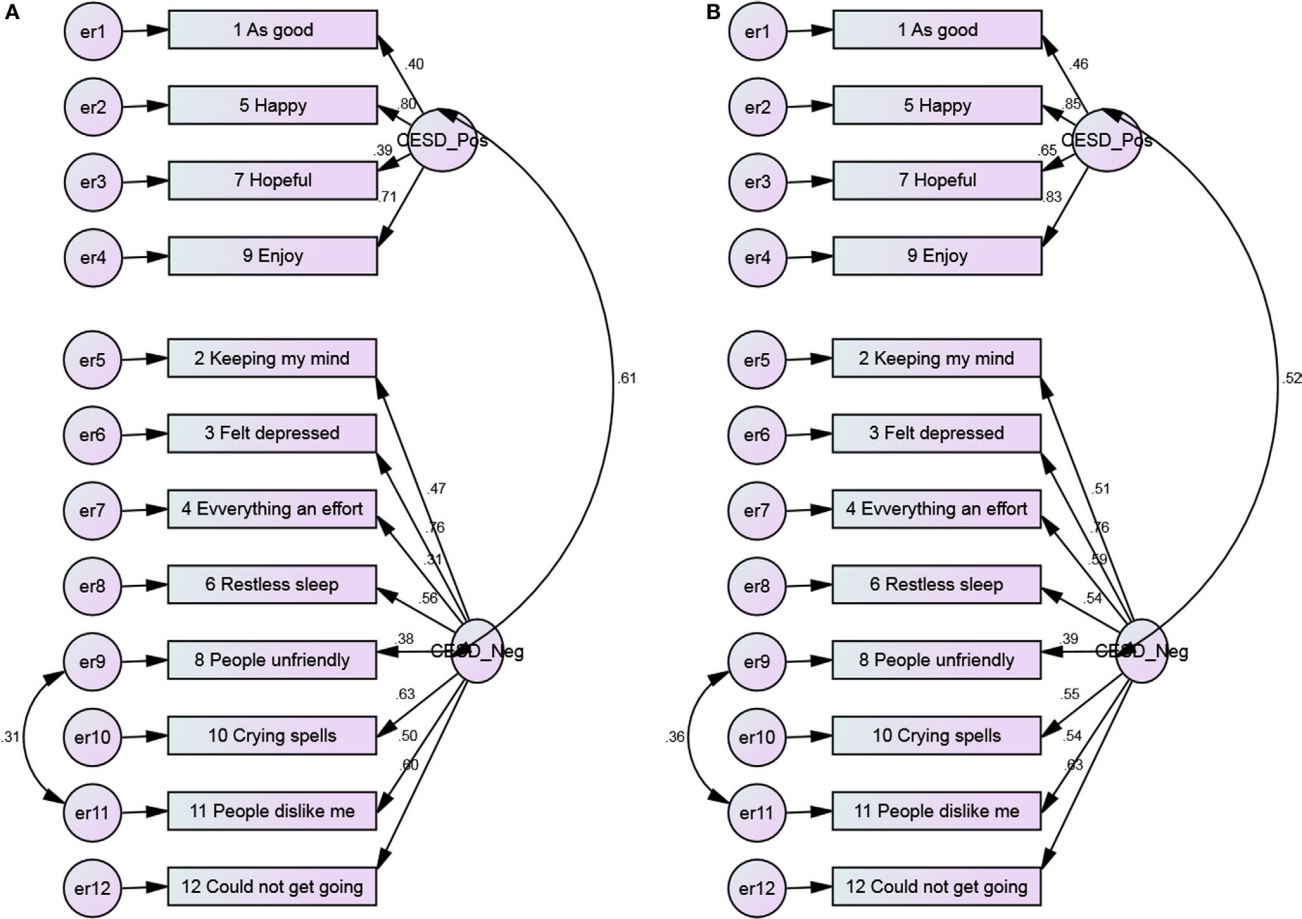
**The 2-factor model of the CES-D 12, with error covariance and no constraints among Blacks and Whites**. **(A)** Blacks and **(B)** Whites.

**Figure 4:**
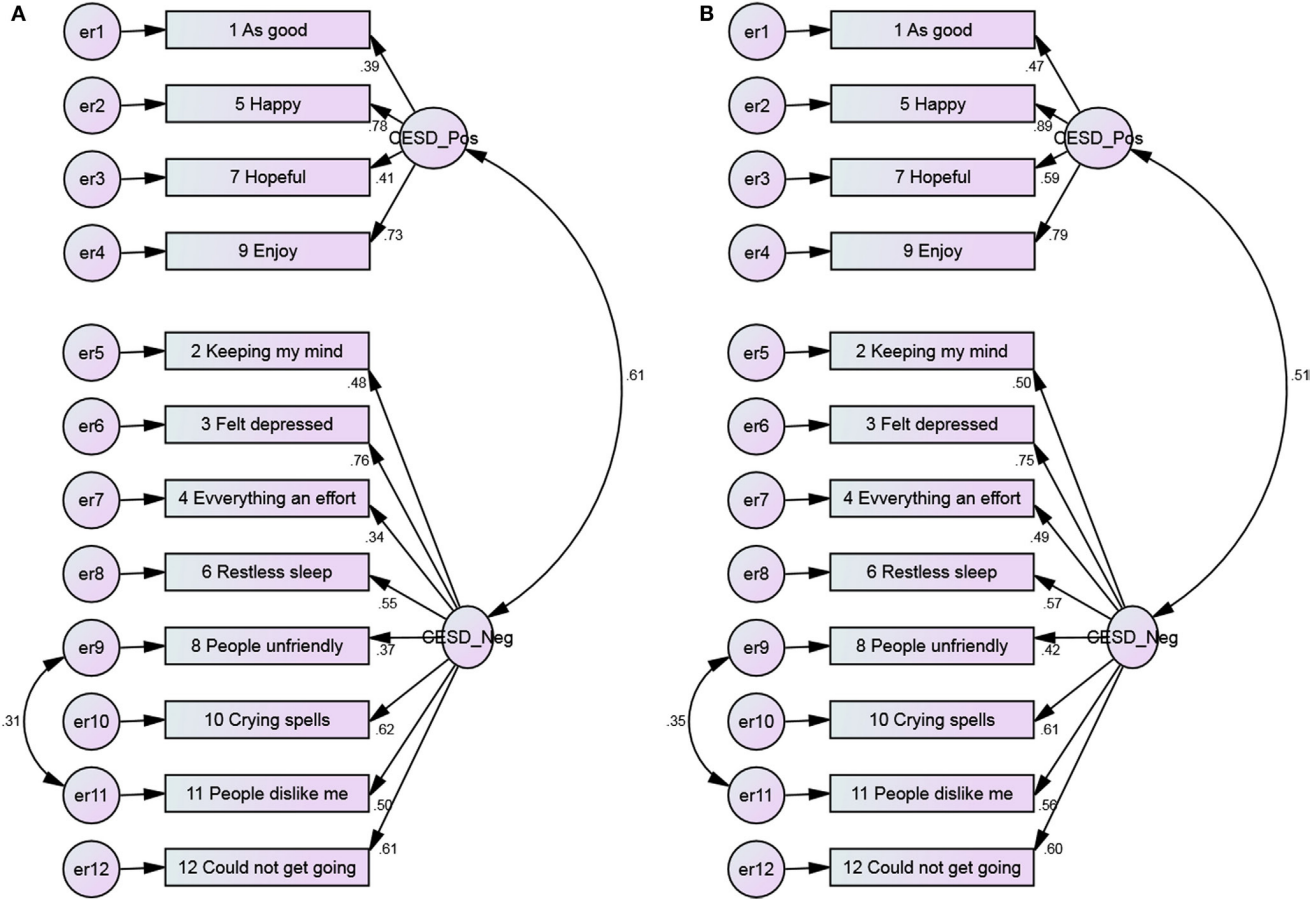
**The 2-factor model of the CES-D 12, with error covariance and constraints among Blacks and Whites**. **(A)** Blacks and **(B)** Whites.

**Figure 5:**
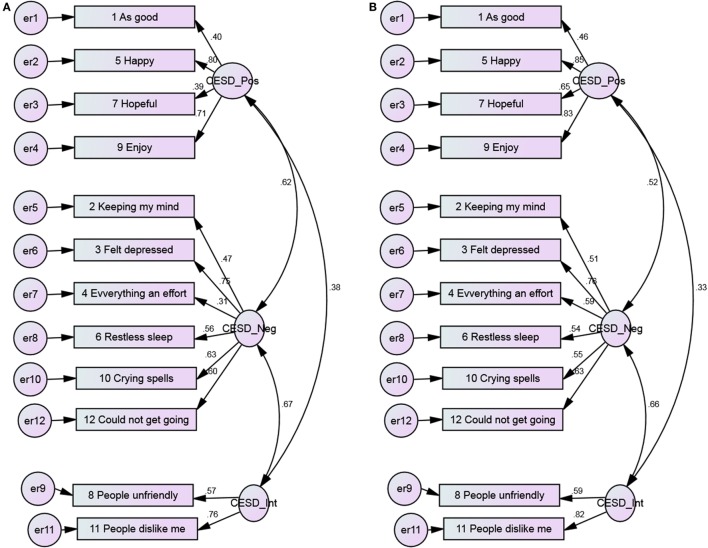
**The 3-factor model of the CES-D 12, with error covariance and no constraints among Blacks and Whites**. **(A)** Blacks and **(B)** Whites.

**Figure 6:**
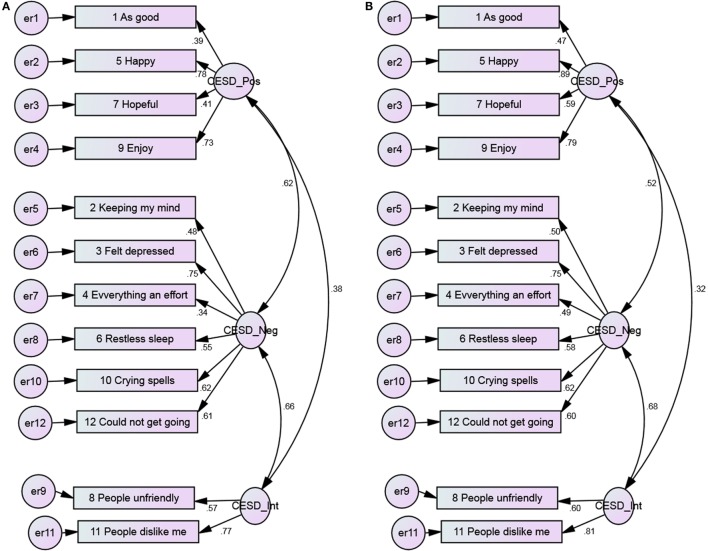
**The 3-factor model of the CES-D 12, with error covariance and constraints among Blacks and Whites**. **(A)** Blacks and **(B)** Whites.

Table 3 represents fit indices for the models. A comparison of chi-square values suggested that fits of the 3-factor models (i.e., *Model 5* and *Model 6*, with correspondent Figure 5 and Figure 6) were significantly better than fits of the 2-factor models (i.e., *Model 3* and *Model 4*, with correspondent Figure 3 and Figure 4). Similarly, fits of the 2-factor models (Figures 3 and 4) were significantly better than that of the 1-factor models (i.e., *Model 1* and *Model 2*, with correspondent Figure 1 and Figure 2).

**Table 3:** **Fit indices of six models with and without constraints and error covariance**.

	Model 1^a^	Model 2^a^	Model 3^b^	Model 4^b^	Model 5^c^	Model 6^c^
**Modeling**						
Factors	1	1	2	2	3	3
Error covariance	+	+	+	+	+	+
Constraints	−	+	−	+	−	+
**Fit indices**						
Chi-square	1081.15	494.77	523.36	573.51	519.94	569.60
Degrees of freedom	96	106	104	114	102	111
Probability level	<0.001	<0.001	<0.001	<0.001	<0.001	<0.001
CMIN/DF	11.26	4.67	5.03	5.03	5.10	5.13
CFI	0.91	0.96	0.96	0.96	0.96	0.96
RMSEA	0.05	0.03	0.03	0.03	0.03	0.03
AIC	1249.15	642.78	675.36	705.51	675.94	707.56
BCC	1250.75	644.18	676.81	706.77	677.42	708.90

*CMIN/DF, chi-square/degree of freedom; CFI, comparative fit index; RMSEA, root mean square error of approximation; AIC, Akaike information criterion; BCC, Browne and Cudeck*.

*^a^Chi-square difference tests showed differences between chi-square values between Models 1and 2*.

*^b^Chi-square difference tests showed differences between chi-square values between Models 3 and 4*.

*^c^Chi-square difference tests showed differences between chi-square values between Models 5 and 6*.

As model fit significantly improved from the 1-factor model to the 3-factor model, the 3 factor model was considered as the optimum solution (chi-square = 596.60, CFI = 0.96, RMSEA = 0.03, *p* < 0.001) for both Blacks and Whites (Table 3). This also suggested invariance of the factor structure between Blacks and Whites.

Model fits significantly changed after imposing constraints to the models, suggesting lack of invariance of the loadings between Blacks and Whites. This pattern was seen for 1-, 2-, and 3-factor models (Table 3).

### Item Loadings for Blacks and Whites

For both Blacks and Whites, the loadings were worst for the 1-factor model and the loading of the item “as good” was very poor. Among Blacks, item “*hopeful*” also had very poor loadings; however, this item had a better loading for Whites. The loadings considerably improved with adding number of factors from 1 to 3.

Positive items had weaker covariance among Blacks compared to Whites (Figures 1 and 2). As Figures 3 and 4 suggest, for 2-factor solution, loading for item “*people unfriendly*” among Whites and loadings for items “*as good*,” “*hopeful*,” “*everything effort*,” and “*people unfriendly*” among Blacks are poor. The covariance of positive and negative factors was slightly higher for Blacks, suggesting that positive and negative factors correlate slightly better in Blacks compared to Whites (Figures 3 and 4). Poor loadings for items “*as good*,” “*keeping mind*,” “*everything effort*,” and “*hopeful*” were seen in the final 3-factor model for Blacks, while the only item with poor loading in this model for Whites was item “*as good*” (Figures 5 and 6).

### Range of Loadings for Each Dimension

In the 1-factor model of the 12-item CES-D scale with constraints and error covariance, item loadings varied between 0.21 and 0.76 for Blacks and 0.25 and 0.73 for Whites (Figure 2).

The 2-factor model of the 12-item CES-D scale with error covariance and constraints ranged from 0.39 to 0.78 (Blacks) and 0.47 to 0.89 (Whites) for positive affect, and 0.34 to 0.76 (Blacks) and 0.42 to 0.75 (Whites) for negative affect (Figure 4).

The 3-factor model of the 12-item CES-D scale, with error covariance and constraints ranged from 0.39 to 0.83 (Blacks) and 0.47 to 0.89 (Whites) for positive affect, 0.34 to 0.75 (Blacks) and 0.49 to 0.75 (Whites) for negative affect, 0.57 to 0.77 (Blacks) and 0.60 to 0.81 (Whites) for interpersonal items (Figure 6).

## Discussion

Through the literature on factor analysis of the CES-D scale among racial and ethnic groups, often invariance of factor structure has been demonstrated while the invariance of loadings has been difficult to establish (24, 37). In other words, factor structure and overall fit of the models are major determinants of measurement equality among racial/ethnic groups and have been subject to invariance in several studies up to now, while the item loadings are subject to more variance due to measurement bias or characteristics of the study sample.

The current study showed invariance for factor structure; however, lack of invariance for item loadings of the 12-item CES-D scale between Blacks and Whites. Although we could not find systematic Black–White differences in the structure of the 12-item CES-D scale, several item loadings were worse among Blacks than Whites. Despite the acceptable fit of our final model, poor loadings were found for more items (i.e., “*as good*,” “*hopeful*,” “*keeping mind*,” and “*everything effort*”) among Blacks than Whites (i.e., “*as good*”).

The invariance of factor structure of the 12-item CES-D scale in this study is in line with CFA for the original 20-item CES-D scale in previous studies, which found the same factor model for Blacks (27), Black women (55, 56), and Black caregivers (57, 58). Torres used the NSAL data and showed that among Black men with Caribbean ancestral ties, CES-D scale scores were not associated with CIDI-based MDD or dysthymia (59). The author found that among Blacks and Black men with Caribbean ancestral ties, the item “I felt that I was just as good as other people” had item-to-total correlations and inter-item correlations below 0.30, and in all groups, the item “I felt like everything I did was an effort” also had item-to-total correlations and inter-item correlations below 0.30 (59); however, Torres did not use CFA and did not include Whites. Thus, our study is not the first to report psychometric limitations of the CES-D scale when applied to multiple racial groups. Previous studies warned that CES-D scale scores should be interpreted with caution in different populations, particularly when comparing scores across racial groups (59, 60).

The results of our CFA for the 12-item CES-D scale indicated invariance of factor structure between Blacks and Whites, with the 3-factor model being the best model among those analyzed. Our results are in line with the recently published paper on exploratory factor analysis of the 11-item CES-D scale (24). Meta-analysis of 4-factor model of the 20-item CES-D scale has previously called into question the appropriateness of such a model for application across multiple racial and ethnic groups (37). On the other hand, CFA of the 20-item CES-D scale among several hundred Black and White women proposed the 2-factor model as the best model (29); however, relatively small sample size and applying models only to the women are among the limitations of that study. Interestingly, in line with our 3-factor model, somatic complaints and depressive affect lack conceptual distinctions in 4-factor model of the 20-item CES-D scale (27). As an explanation, evidence shows that in some cultures, depression may be expressed through somaticized symptoms rather than depressive affect, and somatic complaints in individuals are more associated with experiences of depressive affect rather than positive affect or interpersonal problems (38–40, 44).

The differences in item loadings as well as fit with and without constraints between Blacks and Whites imply lack of invariance of the CES-D scale between Blacks and Whites in terms of items. In comparison to Whites, Blacks showed systematically lower item loadings except for interpersonal problem items. Previous research has documented disproportionately higher endorsement of interpersonal problem items among Blacks compared to Whites (23, 26, 61). These notions imply the need for further studies on measurement equivalence of the CES-D scale among racial and ethnic groups and a need for cross-validating measures of depression such as the CES-D scale with diagnosis of depression based on structured interviews and physician diagnosis.

We found differences for several items between Blacks and Whites, including item “*happy*.” Canady et al. found the item “*happy*” as the only item among the 20-items of original CES-D scale with different loadings between Blacks and Whites after applying the cross-group constraints (29). The sample of the study by Canady et al. was subject to strict matching. Future research should test whether any of these differences in item loadings is a function of socioeconomics or genuine cultural differences in experience and endorsement of depressive symptoms.

Appropriate latent factorial structure of the CES-D scale has implications for clinicians as well as researchers. Putting the items into definite clusters when assessing CES-D scale scores is of clinical importance considering that different ethnic groups may respond to treatments through changes in scores of different symptom clusters (38, 62). Furthermore, it is suggested that Blacks may report more somatic complaints than affect changes due to depression (39), which in turn necessitates both clinicians and researchers to take into account the cultural background of the individuals when interpreting the results of such measures. Future research should be directed toward the reliability of measuring depressive symptoms in different racial/ethnic groups, whether to improve currently available methods or to develop new tools.

This study is subject to several limitations. First, we did not consider potential gender differences in our CFA. Second, similar to most other studies on abbreviated CES-D scales, we did not estimate the fit for the 4-factor model. The reason we did not test the 4-factor model was that we only had 12 items. Third, the difference in sample size between Blacks and Whites may have a potential impact on the results of this study; however, the real extent of this impact is unclear. Among the strengths of this study, which distinguish it from most previous studies, are using a nationally representative sample and large sample size.

In conclusion, the present study indicated invariance of the factor structure for the 12-item CES-D scale in Blacks and Whites. Thus, at least in part, measurement bias associated with using the CES-D scale should be considered when comparing Blacks and Whites for correlates of depression. Further research is warranted to scrutinize the role of socioeconomic and cultural factors that may partially explain Black–White differences in measurement properties of the CES-D scale for depressive symptoms.

## Author Contributions

SA was responsible for the design and analysis of the study. Both authors revised the manuscript and approved the final draft. EM-Z performed a comprehensive literature review and provided the first draft of the manuscript.

## Conflict of Interest Statement

SA and EM-Z declare that they have no competing interests.

## Appendix

**Table A1:** **The 12-Item Center for Epidemiologic Studies Depression (CES-D)**.

CES-D item 1	As good as other people
CES-D item 2	Trouble keeping mind on task
CES-D item 3	Felt depressed
CES-D item 4	Everything an effort includes
CES-D item 5	Felt hopeful
CES-D item 6	Restless sleep
CES-D item 7	Happy
CES-D item 8	People were unfriendly
CES-D item 9	Enjoyed life
CES-D item 10	Crying spells
CES-D item 11	People dislike me
CES-D item 12	Could not get going
